# The single-cell transcriptome and chromatin accessibility datasets of peripheral blood mononuclear cells in Chinese holstein cattle

**DOI:** 10.1186/s12863-023-01139-0

**Published:** 2023-08-07

**Authors:** Xiao Wang, Yahui Gao, Cong-jun Li, Lingzhao Fang, George E. Liu, Xiuxin Zhao, Yuanpei Zhang, Gaozhan Cai, Guanghui Xue, Yan Liu, Lingling Wang, Fan Zhang, Kun Wang, Miao Zhang, Rongling Li, Yundong Gao, Jianbin Li

**Affiliations:** 1 Institute of Animal Science and Veterinary Medicine, Shandong Academy of Agricultural Sciences, Jinan, 250100 China; 2 Animal Genomics and Improvement Laboratory, Henry A. Wallace Beltsville Agricultural Research Center, Agricultural Research Service, USDA, Beltsville, MD 20705 USA; 3 Center for Quantitative Genetics and Genomics, Aarhus University, Aarhus, 8000 Denmark; 4 Shandong OX Livestock Breeding Co., Ltd, Jinan, 250100 China

**Keywords:** Chinese Holstein cattle, Peripheral blood mononuclear cell, Lipopolysaccharide, Single-cell accessibility dataset, Transcriptome, Chromatin

## Abstract

**Objectives:**

This study was performed in the frame of a more extensive study dedicated to the integrated analysis of the single-cell transcriptome and chromatin accessibility datasets of peripheral blood mononuclear cells (PBMCs) with a large-scale GWAS of 45 complex traits in Chinese Holstein cattle. Lipopolysaccharide (LPS) is a crucial mediator of chronic inflammation to modulate immune responses. PBMCs include primary T and B cells, natural killer (NK) cells, monocytes (Mono), and dendritic cells (DC). How LPS stimulates PBMCs at the single-cell level in dairy cattle remains largely unknown.

**Data description:**

We sequenced 30,756 estimated single cells and mapped 26,141 of them (96.05%) with approximately 60,075 mapped reads per cell after quality control for four whole-blood treatments (no, 2 h, 4 h, and 8 h LPS) by single-cell RNA sequencing (scRNA-seq) and single-cell sequencing assay for transposase-accessible chromatin (scATAC-seq). Finally, 7,107 (no), 9,174 (2 h), 6,741 (4 h), and 3,119 (8 h) cells were generated with ~ 15,000 total genes in the whole population. Therefore, the single-cell transcriptome and chromatin accessibility datasets in this study enable a further understanding of the cell types and functions of PBMCs and their responses to LPS stimulation in vitro.

## Objective

Lipopolysaccharide (LPS) composes the outer membrane of Gram-negative bacteria and its exposure can result in elevated levels of local or systemic inflammation in cattle [1], as LPS modulates immune responses as the crucial mediator of chronic inflammation [2]. LPS is a highly potent activator of the innate immune system that can activate cellular responses through the activation of pro-inflammatory transcription factors and binding of TLR4/CD14/MD2 receptor complex to cause strong inflammatory responses in infected animals [3–5]. As the first line of defense, the innate immune system confronts infectious agents through monocytes (Mono), dendritic cells (DCs), granulocytes, phagocytic macrophages, cytotoxic natural killer (NK) cells, and gamma/delta (γδ) T cells [6, 7]. In dairy cattle, peripheral blood mononuclear cells (PBMCs) typically include primary T and B cells, NK cells, Mono, and DCs, but higher levels of γδ T cell receptor (TCR) positive T cells were found in young calves than those of humans and mice [8–10].

Large-scale single-cell PBMC profiling and their responses to LPS stimulation in cattle need to be investigated to determine cell types and functions of PBMCs and further understanding of LPS-mediated bovine PBMC responses regarding the gene transcriptional, chromatin accessibility, and gene-based changes in PBMCs at the single-cell resolution [11]. Therefore, these single-cell transcriptome and chromatin accessibility datasets will permit investigators to summarize the cell types and functions of PBMCs to interrogate complex cellular differentiation, regulations, and interactions when responding to LPS stimulation in vitro.

## Data description

Four whole blood samples were collected from four 2-year-old Holstein female lactating cattle through the tail vein in Jinan Jiabao Dairy Co., Ltd., in Shandong province, China. Afterward, they were mixed in one pool and evenly divided into four replicates. Four whole-blood samples included one control (no LPS) treatment and three case treatments (2 h, 4 h, and 8 h LPS), where 2 µg/ml LPS (Product Number: L2880, Sigma-Aldrich, Saint Louis, MO, USA) was performed at 37 °C. On Hanks’ Balanced Salt Solution (Solarbio; Beijing, China), PBMCs were finally isolated from the whole-blood treated replicates by centrifugation at 500 g for 20 min at room temperature. All experimental procedures were approved by the Animal Ethics Committee of Shandong Academy of Agricultural Sciences, China (No. 20–123). All experimental protocols were approved by the Animal Ethics Committee of Shandong Academy of Agricultural Sciences, China. All methods were carried out in accordance with relevant guidelines and regulations. All experiments were performed in accordance with the ARRIVE guidelines (Animal Research: Reporting of In Vivo Experiments).

Following the user guide of Chromium Single Cell 3’ Reagent Kits v3 (CG000183), reverse transcriptase was mixed with the barcoded isolated cells into the Gel Beads-In-Emulsions (GEMs), where read 1 primer sequence (R1) was put into during its incubation. Via end repair, tailing, adaptor ligation, and PCR procedures, the sample index, P5, P7, and read 2 primer sequence (R2) were included in library construction to generate single-cell RNA sequencing (scRNA-seq) library. Following the user guide of Chromium Single Cell ATAC Reagent Kits v1.1 (CG000209), nuclei suspensions were incubated with transposase to preferential fragment DNA in open regions of the chromatin and adapter sequences were added to the ends of the DNA fragments instantaneously. The single-cell sequencing assay for transposase-accessible chromatin (scATAC-seq) library was constructed via PCR when nuclei were barcoded into the GEMs, where the sample index, P7, and R2 were added. Finally, scRNA-seq and scATAC-seq libraries containing P5 and P7 primers for bridge amplification were sequenced on the Illumina Novaseq 6000 platform (Illumina, San Diego, CA, USA) with double-end 150 bp.

Cell Ranger Single-Cell Software Suite (release 3.1.0) was used to process the raw data, demultiplex raw base-call files into FASTQ files by “mkfastq”, perform alignment, filtering, barcode counting, and unique molecular identifier (UMI) counting by “count”, and align raw reads to the cattle reference genome (ARS-UCD1.2/bosTau9) [12] by “pipeline”. On average, 392,590,940 out of 408,710,340 raw reads were mapped successfully with the mapping rate of 96.05% and 7,689 cells were finally estimated per sample. The cut-off thresholds for quality control include the number of gene expressions per cell between 200 and 3,000; UMI counts less than 200; percentage of mitochondrial-DNA derived gene expression less than 20%. In addition, genes expressed in less than three cells were removed from the datasets. In total, 26,141 single cells with approximately 60,075 reads per cell were generated by scRNA-seq with approximately 15,000 genes including 7,107 (no), 9,174 (2 h), 6,741 (4 h), and 3,119 (8 h) cells for four samples. In the meantime, 3,422 single cells were also were generated by scATAC-seq. All raw data sets and cell-level processed data files of four samples of scRNA-seq and scATAC-seq are listed in Table 1.

This study provided the scRNA-seq and scATAC-seq data at single-cell resolution that could serve as valuable resources for the further understanding of the cell types and functions of PBMCs and their responses to LPS stimulation in vitro.

**Table 1:** Overview of data sets and data files

Label	Name of data sets and data files	File types (file extension)	Data repository and identifier (accession number)
Data set 1	C.scRNA.fastq.gz	*fastq (.fastq.gz)*	NCBI Sequence Read Archive (https://identifiers.org/ncbi/insdc.sra:SRX19487477) [13]
Data set 2	T1.scRNA.fastq.gz	*fastq (.fastq.gz)*	NCBI Sequence Read Archive (https://identifiers.org/ncbi/insdc.sra:SRX19487478) [14]
Data set 3	T2.scRNA.fastq.gz	*fastq (.fastq.gz)*	NCBI Sequence Read Archive (https://identifiers.org/ncbi/insdc.sra:SRX19487479) [15]
Data set 4	T3.scRNA.fastq.gz	*fastq (.fastq.gz)*	NCBI Sequence Read Archive (https://identifiers.org/ncbi/insdc.sra:SRX19487480) [16]
Data set 5	C.scATAC.fastq.gz	*fastq (.fastq.gz)*	NCBI Sequence Read Archive (https://identifiers.org/ncbi/insdc.sra:SRX19487481) [17]
Data set 6	T1.scATAC.fastq.gz	*fastq (.fastq.gz)*	NCBI Sequence Read Archive (https://identifiers.org/ncbi/insdc.sra:SRX19487482) [18]
Data set 7	T2.scATAC.fastq.gz	*fastq (.fastq.gz)*	NCBI Sequence Read Archive (https://identifiers.org/ncbi/insdc.sra:SRX19487483) [19]
Data set 8	T3.scATAC.fastq.gz	*fastq (.fastq.gz)*	NCBI Sequence Read Archive (https://identifiers.org/ncbi/insdc.sra:SRX19487484) [20]
Data file 1	C.scRNA.txt.gz	*text(.tab)*	GEO (https://identifiers.org/geo:GSM7061075) [21]
Data file 2	T1.scRNA.txt.gz	*text(.tab)*	GEO (https://identifiers.org/geo:GSM7061076) [22]
Data file 3	T2.scRNA.txt.gz	*text(.tab)*	GEO (https://identifiers.org/geo:GSM7061077) [23]
Data file 4	T3.scRNA.txt.gz	*text(.tab)*	GEO (https://identifiers.org/geo:GSM7061078) [24]
Data file 5	C.scATAC.txt.gz	*text(.tab)*	GEO (https://identifiers.org/geo:GSM7061079) [25]
Data file 6	T1.scATAC.txt.gz	*text(.tab)*	GEO (https://identifiers.org/geo:GSM7061080) [26]
Data file 7	T2.scATAC.txt.gz	*text(.tab)*	GEO (https://identifiers.org/geo:GSM7061081) [27]
Data file 8	T3.scATAC.txt.gz	*text(.tab)*	GEO (https://identifiers.org/geo:GSM7061082) [28]
Data file 9	Summary.xlsx	*MS Excel file (.xlsx)*	Figshare (10.6084/m9.figshare.22732451.v7) [29]

## Limitations

The pooled blood sample was mixed from four 2-year-old Holstein female lactating cattle in a relatively small sample size, so it is restricted to represent the PBMCs of the whole population of Holstein female lactating cattle.

## Data Availability

The raw data sets and cell-level processed data files were deposited in the Gene Expression Omnibus (GEO) of National Center for Biotechnology Information (NCBI) with the accession number GSE225962 at https://www.ncbi.nlm.nih.gov/geo/query/acc.cgi?acc=GSE225962. The details of raw FASTQ data were showed in Table 1 and references [13–20]. The details of cell-level processed data were showed in Table 1 and references [21–28].

## Abbreviations

***DC***: Dendritic cell
***GEM***: Gel Beads-In-Emulsion
***LPS***: Lipopolysaccharide
***Mono***: Monocyte
***NK***: Natural killer
***PBMC***: Peripheral blood mononuclear cell
***R1***: Read 1 primer sequence
***R2***: Read 2 primer sequence
***γδ***: Gamma/delta
***scATAC-seq***: Single-cell sequencing assay for transposase-accessible chromatin
***scRNA-seq***: Single-cell RNA sequencing
***TCR***: T cell receptor
***UMI***: Unique molecular identifier
